# Deep-Learning-Based Adaptive Advertising with Augmented Reality

**DOI:** 10.3390/s22010063

**Published:** 2021-12-23

**Authors:** Marco A. Moreno-Armendáriz, Hiram Calvo, Carlos A. Duchanoy, Arturo Lara-Cázares, Enrique Ramos-Diaz, Víctor L. Morales-Flores

**Affiliations:** 1 Centro de Investigación en Computación, Instituto Politécnico Nacional, Ciudad de Mexico 07738, Mexico; mam_armendariz@cic.ipn.mx; 2 Gus Chat, Ciudad de Mexico 06600, Mexico; carlos.duchanoy@gus.chat; 3 Escuela Superior de Cómputo, Instituto Politécnico Nacional, Ciudad de Mexico 07738, Mexico; jlarac1300@alumno.ipn.mx (A.L.-C.); eramosd1300@alumno.ipn.mx (E.R.-D.); vmoralesf1300@alumno.ipn.mx (V.L.M.-F.)

**Keywords:** targeted advertising, emotion-based recommendation, augmented reality, computer vision, deep learning

## Abstract

In this work we describe a system composed of deep neural networks that analyzes characteristics of customers based on their face (age, gender, and personality), as well as the ambient temperature, with the purpose of generating a personalized signal to potential buyers who pass in front of a beverage establishment; faces are automatically detected, displaying a recommendation using deep learning methods. In order to present suitable digital posters for each person, several technologies were used: Augmented reality, estimation of age, gender, and estimation of personality through the Big Five test applied to an image. The accuracy of each one of these deep neural networks is measured separately to ensure an appropriate precision over 80%. The system has been implemented into a portable solution, and is able to generate a recommendation to one or more people at the same time.

## 1. Introduction

Today, competition in the market for products and services is intense, so companies have been forced to adopt different strategies to differentiate themselves from the crowd and thereby attract and retain customers [1] because, although the quality of these products or services is an important point, at present the experience that is provided to the user during the acquisition of any product becomes a crucial point. Customizing products or services is a differentiation strategy that allows to satisfy better customer needs [1] to the point that it is associated with a 26% increase in profitability and a 12% increase in the capitalization of the market [2].

Given the importance of differentiating companies, the objective of the system proposed in this work is to display a personalized advertisement for each potential client that passes outside the BubbleTown^®^ establishment, using a screen to display advertising, which will use technologies such as augmented reality to show the user the recommendation and in this way draw their attention. BubbleTown^®^ is a Mexican company with a branch in Mexico City specialized in the sale of customizable tea or yogurt-based drinks.

The objective of the system will be to analyze the client by means of an image of their face and to recommend one of the BubbleTown^®^ products that they might like the most. To achieve the objective, artificial vision techniques will be used from cameras strategically installed in the premises, together with neural networks that will allow estimating the age, gender, and personality of the client.

A recommendation system filters personalized information, seeking to understand the user’s tastes to suggest appropriate things considering the exclusive patterns of them [3]. A content-based recommendation system examines the characteristics of the products in order to identify those that might be of interest to the user. It is common to have product information stored in a database and with the description, together with the user’s profile to generate the recommendation, it is possible to generate a preference profiles for the user’s feedback [4]. For its part, collaborative filtering is the process in which different articles are evaluated or filtered using the opinion generated by users. For its correct operation, the system must have scores or ratings of the article to be recommended, so it requires users to assign ratings to the articles they consume [4].

Through various studies, it has been questioned whether a person’s taste preference is determined by some factor, of which it has been found that age, gender, and even personality can influence these preferences. In [5,6], analysis was carried out considering age, where it was found that young people prefer sweet flavors, while with aging the preference for this flavor reduces, giving way to the preference for salty, sour, and bitter flavors; and regarding gender, in studies such as [7], it has been shown that women tend to prefer sweet flavors 10% more than men, while in [6], it was concluded that men will have greater acceptance towards acidic or bitter flavors. Last but not least, it has also been shown that there is a relationship between personality and the tendency towards some flavor, as is the case of [8] which results in certain personality traits that influence the preference of any kind of flavors.

Until recently, progress in computer vision was based on the features of manual engineering however, feature engineering is difficult, time consuming, and requires expert knowledge of the problem domain. The other problem with hand-designed features, such as background subtraction and edge detection, is that they are too scarce in terms of the information they can capture from an image [9]. Fortunately in recent years, deep learning advances have gained significant attention in fields such as image processing, so the task to obtain data regarding age, gender, and personality will not be handled through traditional techniques, but rather through deep neural networks, algorithms that today have gained importance in the area of computer systems due to their ability to learn.

This work is divided into four parts: The state of the art, methodology, results, and conclusions. It begins by giving a tour of the relevant works that are related to the areas that this work addresses in the section on the state of the art. Afterwards, the methodology section will explain the steps that were carried out to achieve the objective along with a brief explanation of each of them. Finally, in the results section, a short explanation will be given about the most relevant parts at the end of the project.

## 2. State of the Art

Within recommendations, there are many works that propose and achieve the task of recommending a product to a client, but there are few systems whose main focus is the generation of dynamic advertising from the detection of an individual in front of this. The Intel suite^®^ [10,11], distributed in 2011 in the USA, is a targeted advertising device that makes use of automated systems to detect potential consumers through computer vision. Among its most striking features are the use of anonymous sensors that temporarily search and capture patterns of faces or bodies within a predetermined range of vision, in other words, the ability to detect faces; the analysis of anthropometric features so as to provide advertisements through screens, depending on the viewer, is also generated based on attributes such as the age, height, race, and gender of the viewer.

Wang et al., (2020) in [12] use their users’ information, such as age, gender, location, education level, and more to create a personalized recommendation for online courses. On the other hand, some recommenders use deep learning, such as Liu et al., (2019) [13] that presents a recommender which learns from the interaction between user and product through Deep Learning, highlighting the use of convolutional neural networks.

As mentioned in the introduction, this system analyzes the faces of clients to obtain information regarding age, gender, and personality using deep learning. Since 2011, the use of a CNN for estimating age through a face was proposed for the first time [14]. A more recent work, presented by Orozco et al., (2017) [15], uses a neural network with the purpose of obtaining the gender of a person through the image of their face; for this they implemented 2 stages: Generation of candidate regions (ROI) and classification of the candidate regions in the male or female person. Another relevant work is the multi-purpose convolutional network of Ranjan et al., (2017) [16]. This CNN is able to detect faces, extract key points, pose angles, determine smile expression, gender (binary classification), and estimate age, simultaneously. Another work by Xing et al., (2017) [17] carried out a diagnosis on the three types of formulations (classification, regression, or ordinal regression) to estimate age using five cost functions as well as three different multitasking architectures that include estimation age, gender, and race classification. Vasileiadis et al., (2019) [18] proposed a convolutional MobileNet network with TensorFlow Lite, which is suitable for low computational power devices that simultaneously estimates characteristics such as age, gender, race, and eye status, as well as whether the subject is smiling or has a beard, mustache, or glasses. As well as previous works, there are many more that aim to classify, through an image of a face, the age and gender of a person. In addition, other works of value are Zhang et al., (2017) [19], where the faces that appear in a video stream are detected and in [20], Liu et al., (2018), a face detection using LFDNet is presented. In [21], a probability Boltzmann machine network is used for face detection. Zhou et al., (2019) [22] presents a system using the YTF dataset, obtaining a 99.83% correct face detection, and Greco et al., (2020) [23] presented a gender recognition algorithm with a 92.70% accuracy.

Regarding personality, this is not an accessible piece of information that can be found in documents, but rather a characteristic that requires professionals and personalized research in human behavior [24], but it has been discovered that personality traits can be predicted with precision depending on the characteristics of an image, such as the saturation mean, the variation of the value, the temperature, the number of faces, or the color level (Instagram filters) [24].

In 2016, the ChaLearn dataset [25] was created for a contest whose objective was to identify the Big Five in a person through videos, composed of 10,000 videos of people speaking in front of the camera during 15 s obtained from YouTube in 720-p resolution, each tagged by Big Five using Amazon Mechanical Turk. Using deep regressions and convolutional neural networks, the ChaLearn winner combines the results of image analysis (face detection in frames) and analysis of audio characteristics (divided into N pieces) extracted from the dataset videos, to obtain a final mean precision slightly above 91% [24,25].

In [26,27], audio, images (using OpenFace), and spoken text are extracted from the videos in the ChaLearn dataset. In both, there are 3 separate components or channels for processing and extracting characteristics, one for each modality taken, and at the end the results of each component are combined to obtain a personality prediction.

Similarly, the compilation in [28] shows that the precision of jobs where only images are used versus those where they are combined with audio and even text (natural language) varies very little, at no more than 1%, and that the implemented model does not mean a great impact or increase in it.

In [24,29], a new dataset (PortraitPersonality.v2 dataset) was built from ChaLearn’s, which consists of selfie-type images where only one person appears and their face is visible, labeled with the Big Five of the person in the photo. They were tested with the PortraitPersonality.v2 dataset, giving the FaceNet-1 model the best result. FaceNet is a face verification, recognition, and grouping network trained with millions of face images. Applying Transfer Learning reaches an average precision of 65.86%.

### 2.1. Augmented Reality

The use of augmented reality (AR) for advertising and commercial applications lies in completely replacing the need to try anything in stores, thus saving a considerable amount of time for customers, which would probably be used to observe, decide, and select a product (not always concluding in the sale of the same) and thus increase the sales possibilities of the stores [30].

AR also complements web applications by supporting the “live” observation of the objects displayed on screens, as a supplement to what is being produced. Thus, not only is the user informed about when they are “live”, but they can also use it as a learning tool for future activities. In contrast to virtual reality (VR), which creates an artificial environment, AR simply makes use of the existing environment by overlaying new information on top of it. In AR, the information about the surrounding real world is made available to the user for information and/or interaction through the use of screens.

When selecting a beverage from a set of possible options, for example, it is possibly to see it first in your eyes, through a suitable AR application, a virtual glass, which has your preferred beverage with the best tasting quality and other associated characteristics such as the origin of the product, the way the product is processed, the number of calories in a unit of volume, etc.

A study of the market by Grand View Research, the market research firm, points out that this kind of application would generate a considerable increase in sales for stores and restaurants. The total worldwide market for AR is estimated to be more than US$13.4 billion by 2019 and is expected to reach US$340.16 billion in 2028, growing at a CAGR of 43.8% from 2021 to 2028 (www.grandviewresearch.com, accessed on 18 December 2021).

An early start in the realization of the commercial potential of AR was made by the launch of Hololens, a headset capable of creating a virtual vision. This device, with a screen of about one inch by two inches and a thickness of two centimeters, is a product of Microsoft, since its development is carried out in the context of HoloLens, a new project of a company dedicated to the research and development of products focused on augmented reality applications. Perhaps, the best known example is Magic Mirror [31,32], devices that are basically a long-dimensional screen where the customer can interact with various simulated objects, provided by another specific device (markers). The marketing approach used in work [30] is that the users can see their reflection in the Magic Mirror with a virtual model of clothing or a product that they would like to try on. The advantage of this system over going to the store is that once the user selects the garment for testing, they have the ability to change some details, such as color, size, and even stitching.

Another application where augmented reality interacts with the person is Snapchat Lenses [33], which is a popular mobile application that applies filters to the face, such as changing eye color, the shape of the face, adding accessories, having animations started when the mouth is opened or the eyebrows raised, as well as exchanging faces with someone else. Other functionalities are the detection of frontal faces by means of the camera of the mobile device, as well as the application of filters on a three-dimensional mask superimposed on them in real time. A Snapchat and Kohl collaboration [34] resulted in an AR feature that allows customers to visualize Kohl’s products at home within the Snapchat app.

Recently, Berman et al., (2021) [35] published a self-explanatory guide on the following steps to successfully develop an AR app. One of the most important things to consider is how AR will help meet a business’s marketing goals. Regarding selecting channels, wAR can be for online or in-store sales. However, one option to consider is to follow an omnichannel strategy that allows covering all types of customers. Millennials are a good target market for their affinity to new technologies. One last point to highlight is the importance of measuring the return on investment of the AR app, where one crucial aspect is to evaluate AR’s success in increasing profits due to reductions in costs and increased sales.

### 2.2. Related Works

A brief comparison of our work with some published works and industry applications [36] is shown in Table 1. Academic papers focus on facial and gender recognition using various algorithms but do not incorporate other aspects such as Big Five personality analysis, generation of a personalized recommendation, and AR. On the other hand, the AR company apps focus on AR technology, but other elements are missing.

With the review of previous works, it can be said that, although the task of recommending a product to a client has been approached several times, few works do not require having the data of the client’s preferences or history in their database to achieve the recommendation. On the other hand, combining the recommendations with the use of augmented reality is also scarce since it has focused more on other areas such as video games or applications for social networks. In most of the researched works, the similarity is that all the systems are made for previously registered users and that they interact on the commerce website— this limits the use of the recommender to the online user and forgets those who prefer interaction in physical stores, this being the motivation for this work.

The objective of this work is to analyze the face of a client on a particular pose, that is, to show the importance of knowing the client’s personality and their age. For the face recognition of a client, the approach has been made using face detection and classification methods. After this, the work presents the recommendation of products in a commerce display (totem); using the detected client’s age, gender, and personality from the customer’s face, the recommendation is sent by the system allowing to make it possible to use it as feedback to improve the final recommendation.

**Figure 1:**
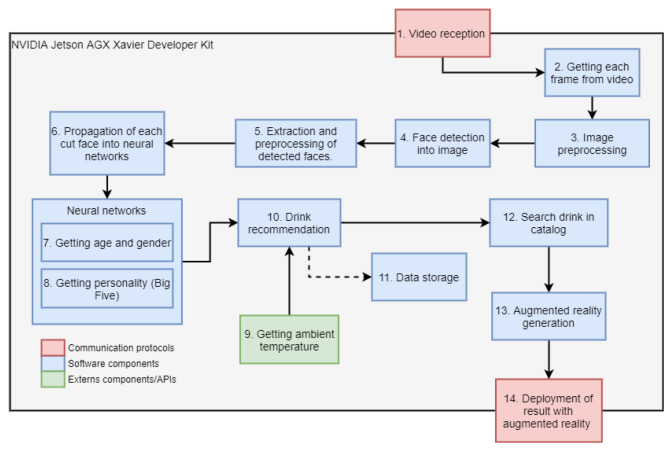
Global system diagram.

The main contributions of this work are:

- A novel deep neural network can predict the age and gender of more than one person in a selfie (see details in Section 3.7).
- A new content-based recommender can select a different beverage for each user.
- A methodology to provide a complete solution to implement an AR system that can be of help to stores that seek to boost sales using an innovative display system (cf. Figure 1).
- A publicity totem that works in soft real-time [49] can present an AR recommendation to the user.

Finally, to our knowledge, a system with all these features has not yet been developed.

## 3. Methodology

The proposed system is composed of deep neural networks that analyze independent characteristics of the user by means of their face (age, gender, and personality), in addition to obtaining the ambient temperature, to be able to be entered into a drinks recommender system and display the recommendation obtained through a module that adds augmented reality to a screen.

Figure 1 shows three types of blocks: The green ones represent external components used for our purposes. Those in red represent actions that are carried out using communication protocols between hardware components, and the blue ones are the modules that were implemented in this work.

Next, a description is made of each of the modules that appear in the diagram in Figure 1, in addition, in the final block used the hardware is explained in a general way.

### 3.1. Video Reception

Video from outside the store is continuously sent from an IP camera to an NVIDIA Jetson device for processing. Previously, the network configuration must be made to communicate all the devices, as indicated in [50].

### 3.2. Video-to-Image Capture

Once the video has been obtained in the NVIDIA Jetson from the camera, it is necessary to extract frame by frame from it, since these are the basis of all the processing, using the OpenCV library.

### 3.3. Image Preprocessing

Four main preprocessing operations were performed on the frame obtained by the previous design:

1. Resize the image: The frames coming from the camera are resized to a size of 300 × 300.
2. Normalize the image Convert the frame to a matrix and then normalize it (divide the maximum value of a pixel in RGB color format by 255).
3. Increase the brightness: Each value of the matrix is increased.
4. Apply the transpose to the image: In order to improve the precision of face detection, the transpose operation is applied to the matrix.

### 3.4. Face Detection in the Image

The architecture of the neural network Single Shot Detection (SSD) [51] has several advantages, such as the ability to detect objects at different scales and resolutions, in addition to performing it at high speed. This is a perfect fit for the needs of the project, as it requires a fast response time and the ability to detect faces at various distances from the camera. Therefore, a Single Shot Detection is used based on a neural network MobileNet [52] to have an even lower processing time.

**Figure 2:**
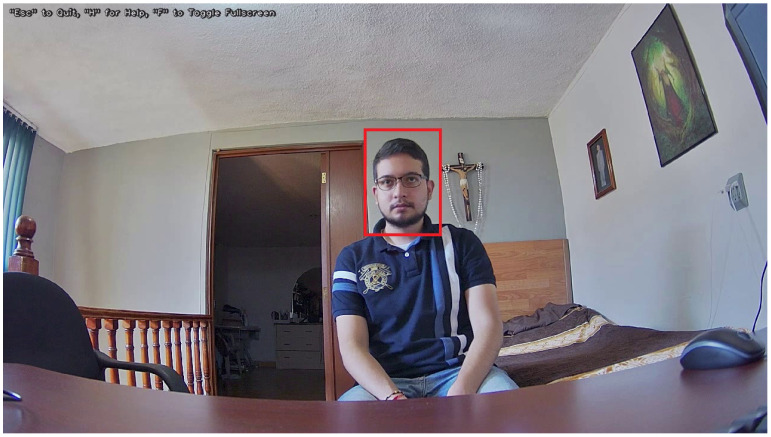
Detected face.

It is necessary to carry out a new custom training to adapt the detection only of faces, so using the API Tensorflow Object Detection [53] that already contains the pre-trained neural network to classify 80 types of classes, together with the transfer of learning technique, this goal is achieved. The datasets used for this process are Face Detection in Images [54] and Google Facial Expression Comparison Dataset [55]. Figure 2 shows an example [55] in which the face is painted in a red box.

### 3.5. Face Extraction and Preprocessing

**Figure 3:**
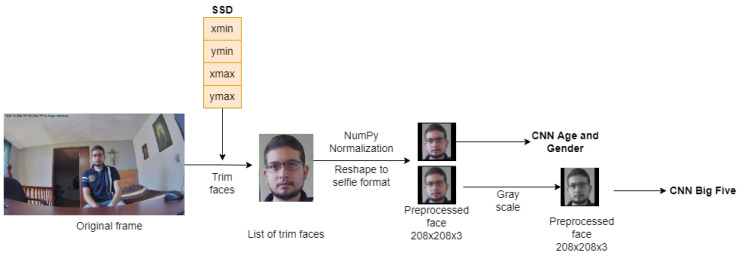
Extraction of the faces detected by the SSD from the frames captured by the IP camera, and their processing to be entered into the neural networks.

Since the location of the faces in the image is known, they are cut out and pre-processed in order to be independently analyze and propagate in a convolutional neural network. This process is illustrated in Figure 3.

### 3.6. Propagation of Each Cropped Face in Neural Networks

**Figure 4:**
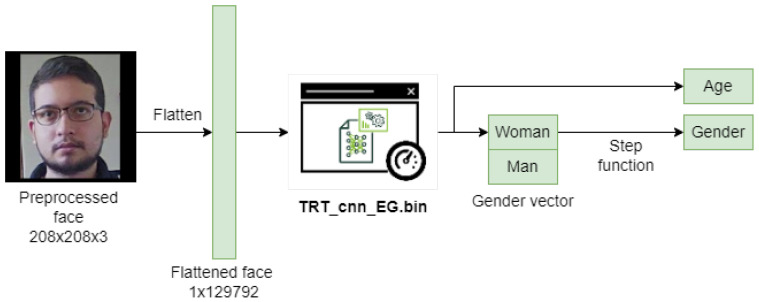
Diagram showing the flattened input face, the age, and gender network model in TensorRT, and its two outputs: The age obtained from the regressor, and the gender vector obtained from the classifier.

**Figure 5:**
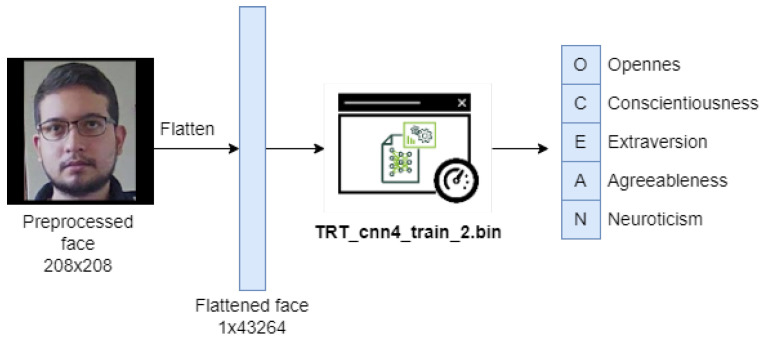
Diagram showing the flattened input face, the classification model: CNN-4 [24] of the Big Five network in TensorRT, and the output vector of size 5 where each position corresponds to a personality dimension as indicated.

Each of the faces obtained in the previous block will be propagated through two convolutional neural networks to estimate age, gender, and personality characteristics. Figure 4 and Figure 5 show these propagations with their respective inputs and outputs.

### 3.7. Getting Age and Gender

**Figure 6:**
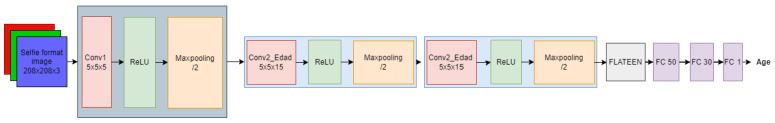
Proposed CNN used to estimate age.

To obtain age and gender from an image of a person’s face in selfie form, a multitasking convolutional neural network is designed with the intention of reducing the amount of computational resources to be used. We start with the layers estimating age, which is the first part of the design. The architecture of this network is shown in Figure 6 and its hyper-parameters in Table 2.

**Figure 7:**
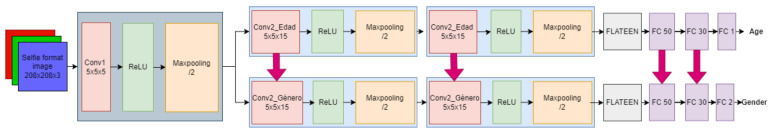
Designed transfer learning.

Afterwards, we carry out a knowledge transfer that will allow us to reduce the training times required to obtain the second CNN that will be responsible for determining the gender of the person. The transfer is carried out in two ways. First, the starting layer enclosed in the gray rectangle (Conv1) freezes during this workout. Second, the final weights of the individual layers are used as initial values for this training which is indicated by the red arrows, see Figure 7.

**Figure 8:**
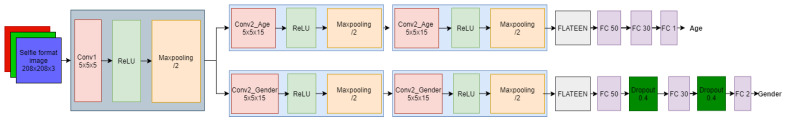
Final architecture of the multitasking convolutional neural network to obtain the age and gender of the person.

Now the training of the second part of CNN is carried out using the hyper-parameters of Table 3. For this training, the regularization of the network was necessary; for this, dropout layers were added. Thus, the final design of the CC is shown in Figure 8.

### 3.8. Obtaining the Personality (Big Five)

The estimation of personality based on his face will be measured using the Big Five model [24], which measures personality through 5 dimensions on scales from 0 to 1:

1. Openness to Experience (O);
2. Conscientiousness (C);
3. Extraversion (E);
4. Agreeableness (A);
5. Neuroticism (N).

For this step, a model previously built and developed from facial analysis in images will be taken, since these provide better results than all types of existing multimedia files (audio, text, video, and images) [27], being the model of classification: CNN-4 of the work in [24,29] the one selected to be integrated into the system, because despite the fact that there is a better model obtained in the same work called FaceNet-1 [56], its weight, computational requirement, and convergence time is very high, causing the selected hardware devices not to be able to support it, taking then the second best model of the work, whose precision obtained does not differ significantly from this, and in return offers better performance and speed. In Table 4 and Table 5, a comparison of the precision in the detection of personality by Big Five is made between the various models of [56] from the images of faces, which have the following characteristics: They are in a selfie format, they are in grayscale, normalized, and their resolution is 208 × 208.

**Figure 9:**
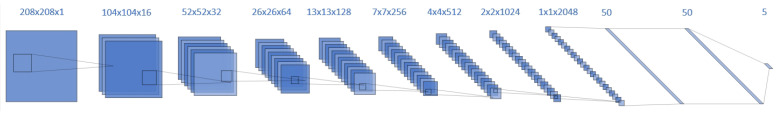
CNN-4 classification model architecture for Big Five [24].

The convolutional neural network architecture of the CNN-4 classification model [56] for the detection of Big Five in the system is shown in Figure 9.

### 3.9. Obtaining the Ambient Temperature

Although the ambient temperature is not a factor that will influence the recommendation of the drink, knowing this information, the system will have the ability to recommend the most suitable drink modality, since within the BubbleTown^®^ catalog there are three options: Zen (hot drink), Iced (cold drink), or Frozen (Frappé). To give the system the ability to obtain the ambient temperature, the API provided by OpenWeatherMap [57] was used.

### 3.10. Drink Recommendation

In very simple terms, a recommendation system is an application that filters information in order to suggest appropriate things to the user [3], which for this job, the recommendation will be a drink from the BubbleTown^®^ catalog.

To achieve the proposed task, this work uses a content-based recommender, that is, a recommendation system that examines the characteristics of the products [4] of BubbleTown^®^ that could be of interest for the user.

The recommender works by using characteristic flavor vectors for each drink on the menu, generated with the support of the *Coffee Taster’s Flavor Wheel* [58] because although there are flavor wheels specific for tea, these wheels have been built considering aroma, texture, and flavor characteristics; while the wheel generated in [58] only considers the flavor.

For the vectorization of the beverages, vectors are first generated for each element of the flavor wheel. This vectorization process is achieved by dividing the wheel into flavor classes (sweet, umami, bitter, sour, and spicy), then the ingredients are listed and a value between 1 (one) and 0 (zero) is assigned depending on the location they have within the flavor class.

With the vectorized basic flavors, the vectors that characterize the ingredients of each drink are added and with the “softmax” function of the resulting vector, what will be the characteristic flavor vector for a said drink is obtained.

**Figure 10:**
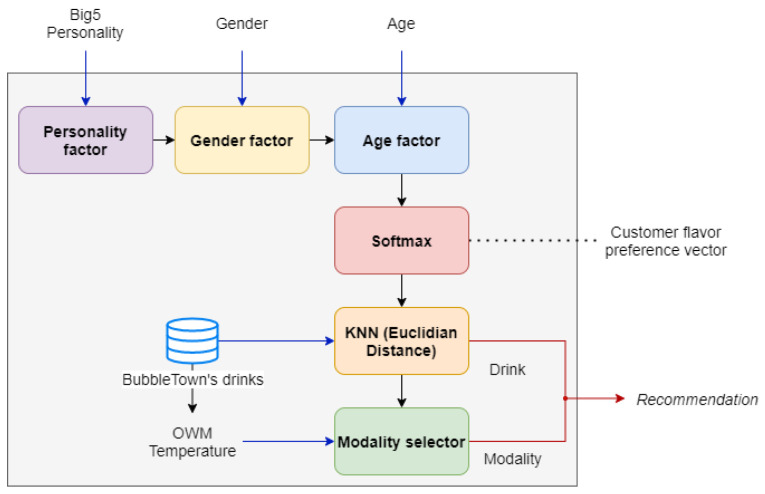
Diagram of the recommender operation.

Figure 10 shows in a general way how the system recommender works. It is important to note that for this recommendation process the salty taste has been eliminated because none of the BubbleTown^®^ drinks have this flavor.

- Personality factor: Once the customer’s personality vector is known together with the Big Five network, the vector will be multiplied by a matrix with the values from Table 6 to obtain a matrix with the customer’s taste preference based on their personality. The next step will be to add all the values for each flavor and thus a vector of size five will be obtained, where each value of the vector will correspond to a flavor class (sweet, umami, bitter, sour, and spicy).
- Gender factor: For this objective, Table 7 is used, so that the vector obtained after applying the personality factor is multiplied by this factor too.
- Age factor: After obtaining the estimated age, the customer will be placed in one of the four age classes and, together with Table 8, the preference vector will be adjusted of the customer’s flavor when multiplied by said table. To continue with the vector format, the flavor *umami* and *spicy* are added however, in the table both have values of 0 (zero), which means that age will not influence these flavor classes.
- Softmax: After applying all the factors that influence the taste preference, the “Softmax” function will be applied to the vector in order to obtain a customer flavor preference vector.
- KNN (Euclidean distance): Since we have the client flavor preference vector, we will use the KNN or “nearest neighbor” algorithm using as a metric, the Euclidean distance to obtain a drink from the database whose flavor is the one that is most similar to that vector.
- Mode selector: From the previous step, you already have the drink that will be recommended by the system. In this step, the drink mode (zen, iced, or frozen) will be selected depending on the ambient temperature.

### 3.11. Data Storage

The system uses a database to store information such as the beverage catalog, the list of static advertising images or the recommendations that the system makes for each person. For this, it was decided to use a non-relational database.

The database will store the drinks that are available for purchase, the static advertising that will be projected when there is no customer in front of the system, and, finally, each of the recommendations generated by the system during its operation. The recommendations are stored together with the recognized face’s features, the estimated age, its gender classification, and the personality vector. The recommended drink will also be stored with said parameters, the ambient temperature at the time of recommendation, and the most suitable drink modality.

Since the face could be considered as a sensitive data, it receives a special treatment before being stored, since it is encrypted using the AES-256 algorithm, in such a way that only system administrators can access this image and only in order to maintain or improve the system proposed through this work.

### 3.12. Search for the Drink in the Catalog

The database contains the characteristic vectors for each drink from the BubbleTown^®^ catalog and the valid modalities for each drink (as explained in Section 3.10) however, the path of the images that will be used to generate the augmented reality is also stored. These images will be loaded into memory in order to create the augmented reality that shows the recommendation to the client.

### 3.13. Generation of Augmented Reality

One of the parts that consumes the most resources is the generation of augmented reality, since to create that feeling of “interaction” with the user it must be constantly updating itself and, considering that at the code level, manipulating the images consists of modifying an array the same size as the image resolution, too many operations are performed to produce a single image. These operations should be carried out approximately 60 times per second if it is to be made an imperceptible process for the user.

**Figure 11:**
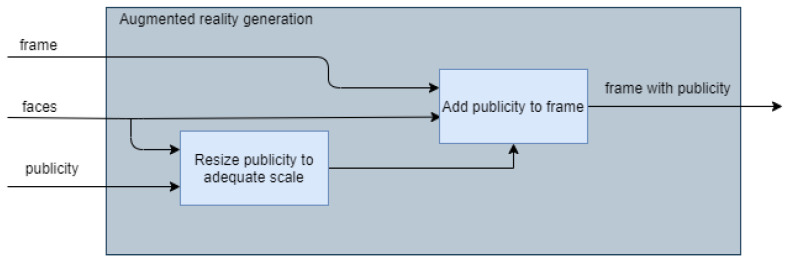
Augmented reality design.

**Figure 12:**
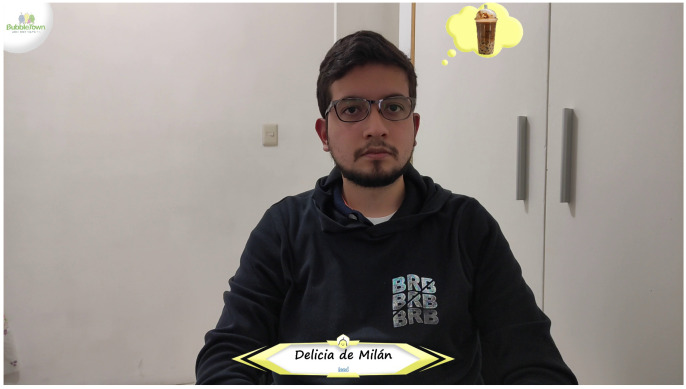
Example of the augmented reality to be carried out.

Figure 11 shows in a general way how the module in charge of generating augmented reality works. To operate the system, it is necessary to feed the module with the frame obtained from the camera, the faces that have been previously recognized and processed, as well as advertising (images of the drinks to be recommended). The first component of the augmented reality module will be in charge of adjusting the size of the thought balloon based on how close or far it is from the camera to create an effect of depth. The next step in this process will be to add the advertising to the original frame, where finally the company logo will be added in the upper left corner, a banner with the name of the drink to recommend at the bottom, and finally, the balloon of thought with the image of the drink in the upper left side of each detected face. In order to exemplify the idea for the reader, the augmented reality proposal is placed in Figure 12.

### 3.14. Display of the Result with Augmented Reality

The image with the recommendation of the drink added in the form of augmented reality is displayed on the LED screen. It is important to mention that periodically, the output on the screen will be refreshed with a new frame (obtained from the camera video).

**Figure 13:**
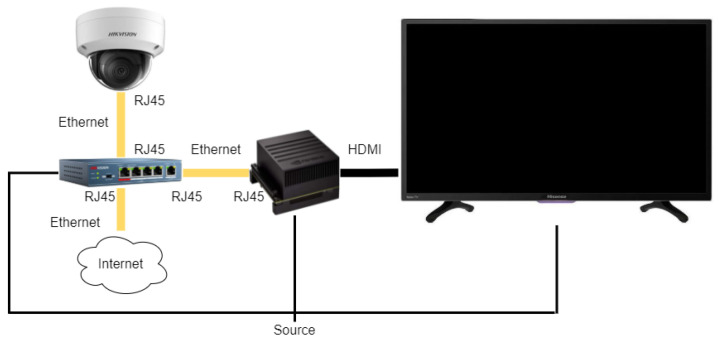
System hardware connection diagram.

At this point, each frame is completely ready to be shown on the screen. The communication flow of the system, as well as the type of information that travels between them, the protocol of communication they use and the way to connect them are shown in Figure 13.

All the functionality will be carried out within the NVIDIA Jetson Xavier, a small but powerful computer for artificial intelligence tasks [59,60] that has an ARM64 architecture and Linux operating system called Jetpack [61].

## 4. Validation

### 4.1. Face Detection

**Figure 14:**
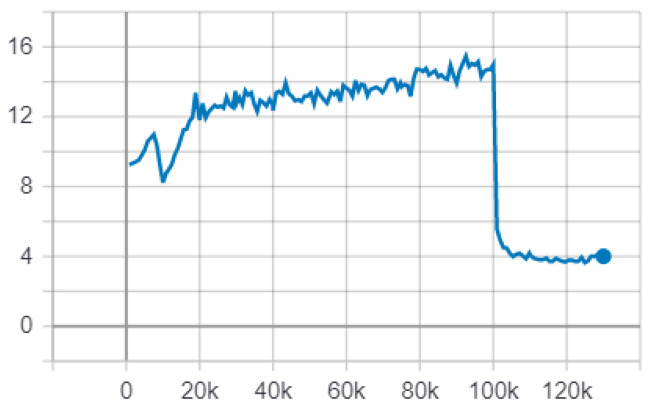
Neural network error Single Shot Detection, note that from epoch 100,000 the error decreases significantly.

The training of the neural network Single Shot Detection was started using only the dataset Face Detection in Images [54] with 100,000 epochs, later, with the aim of reducing the error substantially, 30,000 epochs were trained with both datasets. Figure 14 represents the training of the neural network from epoch 1 to 130,000.

While the error metric is a valid indicative. A confusion matrix allows to know more clearly how the network is performing, since with it, the following scenarios can be considered:

(1) True positive: Is the case where the network detects a face and it is truly a face.
(2) False negative: Occurs when the network detects a face but it is not actually a face.
(3) False positive: Is the opposite case opposite to the false negative, it occurs when the network does not detect a face that should.

To evaluate the three previous cases, the *Intersection over the Union* (IoU) [62] is used as a metric. Table 9 shows the results of the aforementioned confusion matrix.

### 4.2. Obtaining Age and Gender

**Figure 15:**
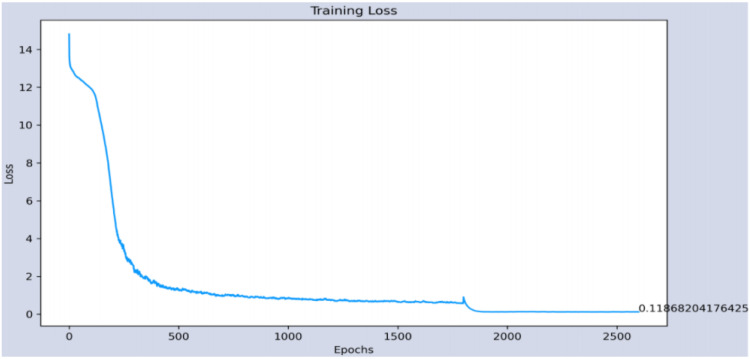
Evolution of the error in the training phase of the age estimation.

**Figure 16:**
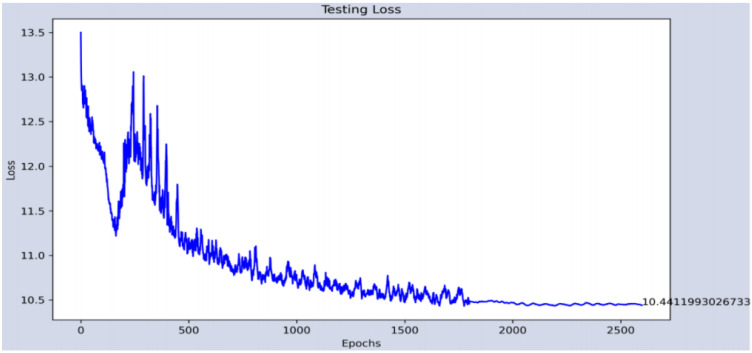
Evolution of the error in the test phase of the age estimate.

The part of the age estimate is measured with the error obtained in the training and testing phases, since it is configured as a regressor. In the training, an average error of 0.11 years is achieved at the end of the 2600 epochs, while in the test set, the average error is 10.44 years. Figure 15 and Figure 16 show the evolutions of the errors during the 2600 training and test periods, respectively.

**Figure 17:**
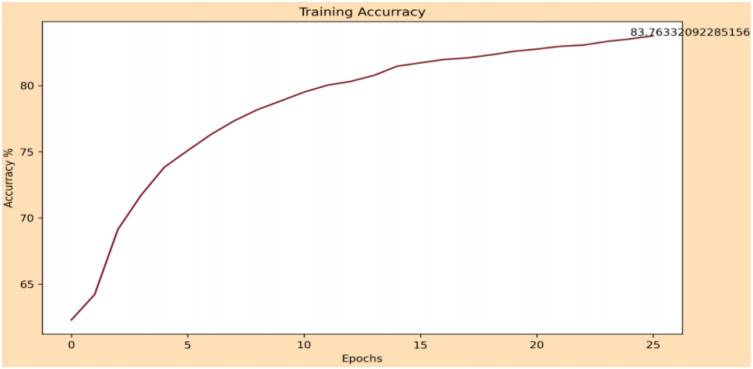
Evolution of precision in the training phase of gender classification.

**Figure 18:**
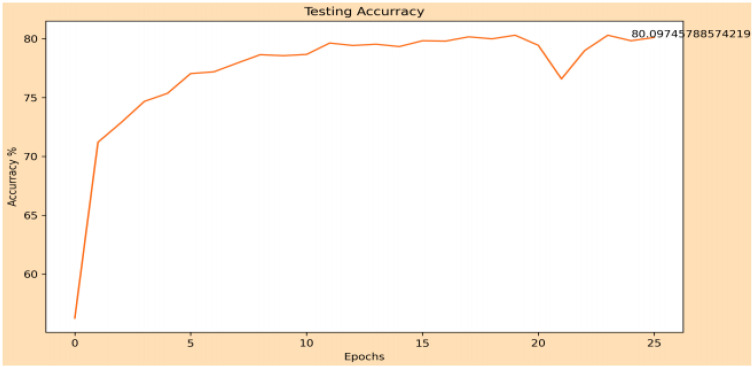
Evolution of precision in the test phase of gender classification.

The classification part of the gender is measured by its accuracy, giving a result of 83.73% in the training phase and 80.09% in the testing phase. The evolution of these are shown in Figure 17 and Figure 18, respectively.

Finally, the confusion matrix of the gender classification is presented in Table 10.

### 4.3. Augmented Reality

**Figure 19:**
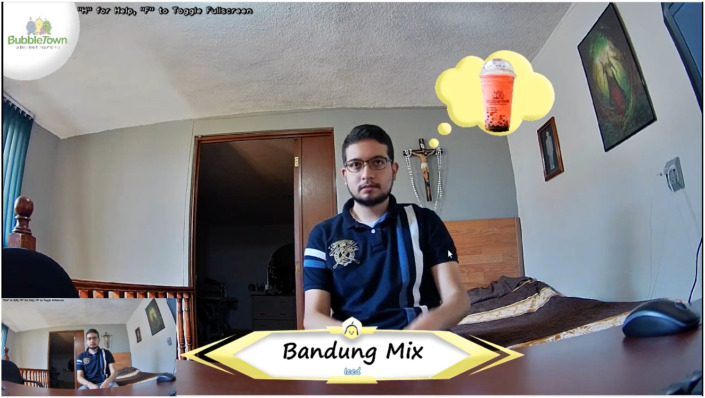
Final result of Augmented Reality.

In Figure 19, the image shown on the screen in a real case is shown. The company logo (Bubble Town^®^ [63]) is displayed at the upper left part of the face, there is the image of the drink and name of the drink and modality are shown in the lower central part. However, even though the NVIDIA Jetson Xavier is a powerful device, it was observed that it is not enough for the project to flow properly, since there is a delay between the video captured by the camera and image displayed on the screen (with augmented reality).

**Figure 20:**
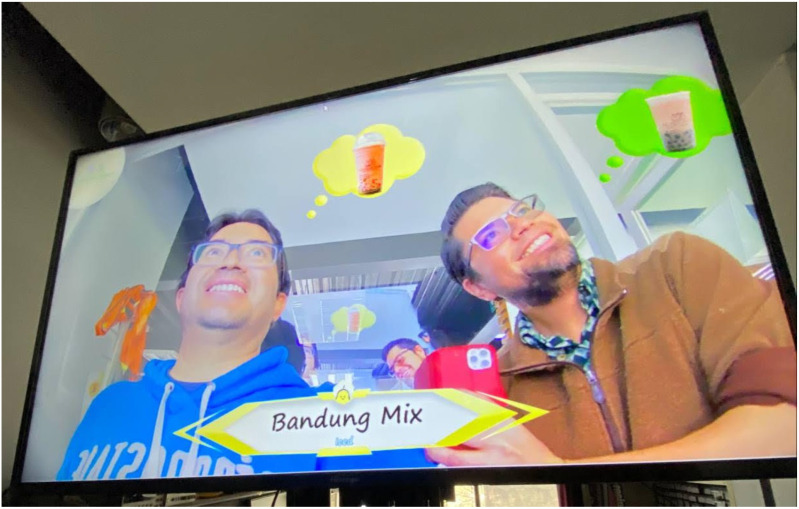
Final result of augmented reality with two people using our advertising totem.

Another important aspect to highlight in the work is the capacity of the system to be able to generate a recommendation to more than one person at the same time, see Figure 20, given that if there are several faces in the camera image, the system will recognize them and generate recommendations independently. So that each user knows the recommended drink, the thought balloons for each one, and the banner will be changing from time to time; to identify who the banner belongs to, it will have the same color as the thought balloon. The advertising totem was in operation for several days in the Laboratory of Computational Cognitive Sciences of the Center for Computing Research [64], showing that it properly worked at all times. To replicate these results, the reader can visit https://github.com/vicleo14/PublicidadBT (accessed on 20 December 2021). A short video demo can be found at https://tinyurl.com/2p8bf68s (accessed on 20 December 2021). Regarding working time, once the system detected a face, it started generating the augmented reality scene in 2.03 s on average; lastly, on a small poll with 30 users, 86.66% of them liked our beverage recommendation; see all data in Table A1 and logifle in https://tinyurl.com/59ev279p (accessed on 18 December 2021).

## 5. Conclusions

Generating targeted, different, and personalized advertising to recommend a product to the customer in an unconventional way is not a trivial task. The main reason that customer tastes can become so complex is that they become unique and unrepeatable preferences; for this reason, achieving generalization of the entire process within the system is highly complex in terms of development and effectiveness.

The raw material of this work are the images in which the clients appear. The preprocessing of the images that will be entered into the deep learning models is one of the main aspects that will influence obtaining good results, since having an adequate processing in the data will allow the model to obtain a result with high precision using fewer resources.

The personalized retraining of the Single Shot Detection with the help of transfer of learning (in order to achieve good results without the need for extensive training) and its results, as well as the execution tests of the convolutional neural network to estimate age and gender and the neural network for Big Five confirm something that is very clear in the world of artificial intelligence: No model will be completely accurate in scenarios of the real world. There is no model that is perfect, that is why the field of deep learning has had periods of oblivion throughout history, and although recently, thanks to more powerful computers, as well as large data banks, it has been a relevant of study, since precisely obtaining an ideal model is one of the objectives that these fields seek to achieve.

With all this, face detection has an acceptable performance for the purposes of the system, although it could be improved, since it must be observed that the almost null existence of data sets available for commercial use or with free licenses is the main cause of not being able to refine or perfect the Single Shot Detection to detect faces in very difficult conditions, such as, people with glasses (whether dark or transparent), with hats, scarves, with tied hair in the case of women, and recently with a mouth mask. The recommendation generated by the system, in the end, is a suggestion based on certain parameters identified in a person, and clearly the ultimate decision to accept or reject it will be with the clients. The importance of this works lies in the “aggressiveness” in which it is suggested, and since it is simply a graphic that does not compromise the decision or intentions of the buyer, in addition to how attractive augmented reality can be for a public unfamiliar with this technology, it is considered to be more likely to arouse interest in Bubble Town^®^beverages rather than having some rejection or negative impact due to a breach of their personal data.

A complementary part of this system is to take into account the need to safeguard people’s sensitive data (faces), stored in its database, to comply with business rules, and not incur any violations to Federal Law on the Protection of Personal Data. For this reason, a privacy notice is provided along with the system, and information that could be considered sensitive is encrypted to prevent its misuse. 

## Figures and Tables

**Table 1:** Related works in the literature and industry.

Authors	Face/Gender	Big5	Ambient	Product	Augmented	Advertising
	Recognition	Personality	Temperature	Recommendation	Reality	Totem
Kim et al., (2021) [37]	□	□	□	□	*√*	□
Kohls (2020) [34]	□	□	□	□	*√*	□
Xueyi et al., (2020) [21]	*√*	□	□	□	□	□
Wayfair (2020) [38]	□	□	□	□	*√*	□
Ikea (2019) [39]	□	□	□	□	*√*	□
Adidas (2019) [40]	□	□	□	□	*√*	□
L’Oréal (2019) [41]	□	□	□	□	*√*	□
Zhou et al., (2019) [22]	*√* *√*	□	□	□	□	□
Asos (2019) [42]	□	□	□	□	*√*	□
Hamid et al., (2018) [43]	□	□	□	□	*√*	□
Liu et al., (2018) [20]	*√*	□	□	□	□	□
Zara (2018) [44]	□	□	□	*√*	*√*	□
Hamid et al., (2016) [43]	□	□	□	*√*	*√*	□
Sephora (2016) [45]	□	□	□	□	*√*	□
Zhang et al., (2017) [19]	*√*	□	□	□	□	□
Lacoste (2014) [46]	□	□	□	*√*	*√*	□
Herm$\overset{‘}{e}$s (2015) [47]	□	□	□	□	*√*	□
Converse (2012) [48]	□	□	□	□	*√*	□
Ours	*√* *√*	*√*	*√*	*√*	*√*	*√*

**Table 2:** Hyper-parameters used to train age in a CNN.

Hyperparameter	Value
Epochs	2600
Learning Factor	1 × 10${}^{-4}$
Batch Size	4

**Table 3:** Hyper-parameters used to train the genre rating portion of CNN multitasking.

Hyperparameter	Value
Epochs	25
Learning Factor	1 × 10${}^{-4}$
Batch Size	4

**Table 4:** Comparison of the best results obtained from the different Big Five models developed in [56].

Model	Average Precision
Regressor: ACoder	50.05% (with tolerance)
Regressor: CNN-2 (B)	52.06% (with tolerance)
Clasiffier: CNN-4	**65.77**%
Classiffier: FaceNet-1	**65.86%**

**Table 5:** Comparison between the results (each one is the percentage of precision of the detection of each personality) by dimension of personality among the best Big Five models in [56].

Model	Precision	O	C	E	A	N
Human	56.6	58.3	50.0	33.3	**83.3**	58.3
CNN-4	65.7	62.0	67.9	72.1	61.4	65.2
FaceNet-1	**65.8**	**61.4**	**69.5**	**73.2**	60.6	**64.3**

**Table 6:** Average taste preference as a function of personality in values from 0 to 1. Table obtained with data from [8].

Taste Preference	O	C	E	A	N
Sweet taste	0.7243	0.4357	0.6138	0.5614	0.4036
Umami taste	0.2986	0.4900	0.5852	0.1886	0.1979
Bitter taste	0.2786	0.1307	0.1600	0.2424	0.1350
Acid taste	0.0271	0.1264	0.2381	0.0252	0.1543
Spicy taste	0.4857	0.2421	0.5971	0.6424	0.5607

**Table 7:** Average taste preference based on gender. Table obtained with data from [8].

Taste Preference	Female	Male
Sweet taste	0.7789	0.7292
Umami taste	0.4434	0.3752
Bitter taste	0.2530	0.3596
Acid taste	0.3783	0.4472
Spicy taste	0.5431	0.8288

**Table 8:** Average taste preference based on age group. Table obtained with data from [6].

Taste Preference	1–17 Years	18–36 Years	37–50 Years	>50 Years
Sweet taste	0.9520	0.8500	0.8100	0.7700
Umami taste	0.0000	0.0000	0.0000	0.0000
Bitter taste	0.2652	0.2400	0.1867	0.1700
Acid taste	0.1972	0.2033	0.2067	0.2233
Spicy taste	0.0000	0.0000	0.0000	0.0000

**Table 9:** Confusion matrix for face detection.

	Positive	Negative
**True**	6818	-
**False**	830	2617

**Table 10:** Gender classification confusion matrix.

	Female (target)	Male (target)
**Classified as Female**	5094	1455
**Classified as Male**	1088	5295

## Data Availability

The authors are committed to providing access to all the necessary information so that readers can fully reproduce the results presented in this work. For this, all the necessary information is available in the following repository at https://github.com/vicleo14/PublicidadBT (accessed on 20 December 2021). A demo is also available at https://tinyurl.com/2p8bf68s (accessed on 20 December 2021).

## Appendix A

**Table A1:** Results of the elapsed time to generate the AR and the satisfaction survey.

Poll Number	Elapsed Time (s)	Did You Like the Recommendation?
1	1.479222298	like
2	1.285918808	dislike
3	1.27488842	like
4	2.368936348	like
5	2.293576145	like
6	2.358893967	like
7	2.369532681	like
8	2.393731785	like
9	2.377891922	like
10	2.281593037	dislike
11	2.267432785	dislike
12	2.641290951	like
13	2.449003696	like
14	2.427659225	like
15	2.363572884	like
16	2.310142899	like
17	2.294511223	like
18	1.219994068	like
19	2.308717346	like
20	2.259387589	like
21	2.253286648	like
22	2.304634762	like
23	1.212442875	like
24	1.298417091	like
25	2.355474758	like
26	2.696813393	like
27	1.217700481	like
28	1.231303215	dislike
29	1.212442875	like
30	2.29670248	like
Average:	**2.036837222**	**86.66%**
